# Prophylactic impact of nano-selenium on performance, carcasses quality, and tissues’ selenium concentration using reversed-phase high-performance liquid chromatography during microbial challenge in broiler chickens

**DOI:** 10.14202/vetworld.2020.1780-1797

**Published:** 2020-09-04

**Authors:** Essam S. Soliman, Fadwa F. Mahmoud, Mai A. Fadel, Rania T. Hamad

**Affiliations:** 1 Department of Animal Hygiene, Zoonosis and Animal Behavior, Faculty of Veterinary Medicine, Suez Canal University, Ismailia 41522, Egypt; 2 Food Hygiene and Microbiology, Reference Laboratory for Veterinary Quality Control on Poultry Production, Animal Health Research Institute, Agriculture Research Center, Ismailia 41511, Egypt; 3 Pharmacology and Pyrogen Unit, Chemistry and Food Deficiency Department, Animal Health Research Institute, Agriculture Research Center, Dokki, Giza 12618, Egypt; 4 Department of Pathology, Faculty of Veterinary Medicine, Menoufia University, Al Minufya 33511, Egypt

**Keywords:** broilers, carcass, immunity, nano-selenium, performance, reversed-phase high-performance liquid chromatography

## Abstract

**Background and Aim:**

Nano-selenium (NS) supplementation contributes in improving productivity, performance, and meat quality while reducing public health concern. Influence of NS and inorganic selenium (Se) water additive on performance, carcass quality, immunoglobulin concentration, intestinal microbiota, Se tissue concentrations, and tissue architecture was studied.

**Materials and Methods:**

Two-hundred and sixty 1-day-old Hubbard chicks were randomly grouped into five groups (5×52) and supplemented with 0.5 and 1.0 mL of NS and inorganic Se (100 mg.L^−1^). G1, G2, G3, and G4 were challenged with *Escherichia coli* O157: H7 2.6×10^8^ on the 14^th^ day. A total of 2250 samples, including 250 sera, 250 intestinal swabs, and 1500 organ and tissue samples as liver, spleen, heart, bursa, intestine, and breast muscles, and 250 eviscerated carcasses were collected.

**Results:**

The results revealed a highly significant increase (p<0.01) in live body weights, weight gains, performance indices, carcasses, and organs weights, whereas immunoglobulin G and M concentrations in broilers treated with 0.5 and 1.0 mL NS, respectively, synchronized reveal a highly significant decline (p<0.01) in total bacterial and *Enterobacteriaceae* counts of intestinal swabs and breast muscles, final pH_24_, and drip loss in broilers treated with 0.5 and 1.0 mL NS, respectively. Meanwhile, water holding capacity revealed no significant differences between all groups. Reversed-phase high-performance liquid chromatography examination revealed the earlier disappearance of NS residues than inorganic Se from the broiler’s liver and muscles. Histopathological photomicrographs of the liver, spleen, bursa of Fabricius, and intestine, as well as, the immunohistochemistry of intestinal sections revealed superior tissue architecture in broilers treated with NS contrary to inorganic Se.

**Conclusion:**

The study showed significant stimulation actions of NS on performance, immunity, carcass and meat quality, intestinal and muscles’ bacterial load as well as short withdrawal period and nearly normal cellular architecture compared to inorganic Se.

## Introduction

The poultry industry in the past 10-20 years has been witnessing a large number of feed additives that enhance broiler’s productivity and survival Moreover, they were recommended among preventive and biosecurity measures in broiler’s farms for their known actions as immune-stimulants. These additives include probiotics [1], synbiotics [2], *Nigella sativa* Linn [3], organic acids [4], and trace elements like selenium (Se) [5].

Se has been widely used in the poultry industry for their organic forms (selenomethionine) that can be easily absorbed by red blood cells by means of active transport [6]. Se after absorption and when not metabolized helps in protein synthesis in the liver, kidney, muscles, and gastrointestinal tract [7]. Since Se supplementation is able to improve the broiler’s productivity and performance, the main concern has also been in the improvement of meat quality and reduces public health concern [8]. Se is also an essential micronutrient because of its unique antioxidant properties [9]. Nano-Se (NS), on the other hand, has been considered a potential supplement for broilers to acquire high catalytic efficiency, low toxicity, and antibacterial activity [10,11]. NS water supplementation at lower doses and concentration (0.5 mL of NS solution at a rate of 100 mg.L^−1^ per each 1-l drinking water) proved to superiorly and significantly improve growth traits, behavior, carcass quality, immunoglobulin concentrations, the histopathological architecture of some organs and tissues (liver, heart, bursa of Fabricius, and spleen), and intestinal microbial load in Arbor and Ross broiler breeds in the presence of heat stress conditions compared to inorganic Se [12]. Se bioavailability is dependent on many factors such as intestinal absorption and biological activation [13], this Se is mostly deposited in muscles, liver, and plasma [14] compared to NS that proved to be less toxicity and high bioavailability in broilers.

High-performance liquid chromatography (HPLC), known as high-pressure liquid chromatography, is a popular analytical technique used for separation, identification, and quantification. HPLC is an advanced technique of column liquid chromatography. The solvent usually flows through a column by gravity but in the HPLC technique, the solvent is forced under high pressures up to 400 psi so that the sample can be separated into different constituents by the difference of relative affinities [15]. HPLC is used increasingly in the analysis of food samples to separate and detect additives, antioxidants, and contaminants. This method breaks down complex mixtures into individual compounds, which, in turn, are identified and quantified by suitable detectors and data handling systems [16]. The previous works established and optimized analytical methods to identify and quantify seleno-amino acids and inorganic Se in biological samples by reversed-phase (RP) chromatography [17].

The study aimed at evaluating the influence of two different doses (0.5 and 1.0 mL) of NS (Green synthesized NS) and commercial inorganic Se (sodium selenite, EMIC Veterinary) at a rate of 100 mg.L^−1^ as a water additive on productive performance, carcass quality (carcass weights and immune organs’ weights), meat quality (pH, drip loss, and water holding capacity), immunoglobulin concentrations, intestinal and muscular microbial counts (total bacterial and Enterobacteriaceae counts), tissue and organs architecture (liver, bursa of Fabricius, spleen, and intestine), and tissue Se (NS and Se) concentrations in liver and muscles of Hubbard broilers challenged with *Escherichia coli* O157: H7 2.6×10^8^ at the 14^th^ day of age.

## Materials and Methods

### Ethical approval

The guidelines used for the care and use of birds were approved by the Scientific Research Ethics Committee, Faculty of Veterinary Medicine, Suez Canal University, Egypt, with approval number (2020005).

### Study period and location

The experimental study was conducted from 17-09-2018 to 25-10-2018. The study was carried out in the poultry houses experimental units located in the Faculty of Veterinary Medicine, Suez Canal University, Ismailia, Egypt. Performance, carcass quality, immunological, and bacteriological examinations were conducted in Animal, Poultry, and Environmental Hygiene laboratories, Ismailia. Meat characteristics were conducted in Food Hygiene And Microbiology Laboratories, Reference Laboratory for Veterinary Quality Control on Poultry Production, Ismailia. RP-HPLC and electron microscopy were conducted in Pharmacology and Pyrogen Unit Laboratories, Chemistry and Food Deficiency Department, Giza. Histopathology and immunohistochemistry were conducted in Pathology Department laboratories, Al Minufya.

### Experimental birds grouping and design

The experiment was designed for a multifactorial randomized control trial. A total of 260 1-day-old Hubbard chicks were purchased from Alexandria Poultry Company and housed on a deep litter system (wheat hay). The litter was treated before the birds’ arrival using sodium metabisulfite (0.05 g/m^2^) as recommended by Soliman *et al*. [18] to control and optimize litter abiotic conditions that when mishandled might lead to the growth and multiplication of pathogenic microorganisms, in addition to minimizing moisture and ammonia in the house microclimate. Chicks were randomly grouped into five groups; each group consisted of 52 chicks (four replicates of 13 birds).

### Broiler’s management and feeding

Birds were brooded on their arrival to a rearing site of 34°C temperature. The temperature was gradually decreased by 3.0-3.5°C weekly until achieving a thermo-neutral zone ranged from 21 to 26°C by the end of the 3^rd^ week. Ventilation was served by both natural (V-shaped windows) and artificial means (supplying fans) to enhance the broiler’s microclimatic conditions and to maintain the natural convection act (Stack effect). A continuous lighting regimen of 23 h lighting and 1-h darkness was provided using an 18 watt-1750 lumen LED white lamps as recommended by Soliman and Hassan [19]. Birds were supplemented with a standard corn-soybean basal diet to satisfy their nutritional requirement, according to the National Research Council [20] and Applegate and Angel [21] modifications. The ration was based on 23% crude protein, 4.8% fat, 3.85% crude fiber, and 3050 kcal/kg energy in the starter rations that were provided from day 1 until the 15^th^ day, while grower ration was provided from the 16^th^ day ongoing for the remaining time based on 21% protein, 5.3% fat, 3.1% crude fiber, and 3100 kcal/kg energy. The ration provided was supplied with vitamin and mineral mix that did not include any of the Se forms as requested to prevent the interaction with the water treatment used in the experiment. The vitamin and mineral mix included Vitamin A 4,000,000 IU, Vitamin D3 1,000,000 IU, Vitamin E 50 IU, Vitamin K 32,000 mg, Vitamin B1 1500 mg, Vitamin B2 7000 mg, Vitamin B6 2500 mg, Vitamin B12 13 mg, Biotin 110 mg, niacin 30,000 mg, calcium pantothenate 10,000 mg, folic acid 2500 mg, manganese 100,000 mg, iodine 1000 mg, iron 35,000 mg, and copper 12,000 mg. The birds were given *ad libitum* access to water. Mortalities, temperature, and relative humidity were recorded daily during the experiment that was designed to last for 39 days.

Birds were subjected to mass active artificial immunization act using drinking de-chlorinated water against infectious bronchitis using live attenuated virus vaccine (PESTIKAL B1 SPF 1000 dose, H120 ≥10^3.5^ EID_50_/dose) at 7^th^ day, infectious bursal disease using live attenuated virus vaccine (SER-VAC D78 Strain 1000 dose, VMG91 ≥10^3.0^ TCID_50_) at 13^th^ and 20^th^ days, and Newcastle disease using live lentogenic virus vaccine (PESTIKAL 1000 dose, Lasota ≥10^6.0^ EID_50_) at 18^th^ and 28^th^ days.

### NS and Se supplement

NS were prepared as recommended by Gorer and Hodes [22] and Ali *et al*. [23] using Se powder (Merck 209651, 100 mesh, ≥99.5% trace metals basis) and sodium sulfate (Merck 7757-82-6, Molecular Weight: 142.04), as well as, glucose 6% powder (Merck G7021, powder, BioReagent, ≥99.5%) as stabilizing and reducing agent, and polyvinyl alcohol (PVA 10,981, mounting medium with DABCO^®^, 100 mL) to prevent deviations in the morphological characters of NS and to protect the molecules from aggregation. The aqueous sodium seleno-sulfate solution was prepared by adding Se powder to sodium sulfate solution, and then optimized by refluxing and heating at 70°C; the filtrate of the formed solution was treated with glucose and PVA 1% with refluxing for an extra 6 h. The final product was examined and identified for its size, concentration (100 mg.L^−1^) and structure using HR-Transmission Electron Microscopy (TEM) JOEL JEM-2010 operated at an accelerating voltage of 200 kV equipped with Gatan digital camera Erlangshen ES500 by dropping the sample on a copper grid coated with a carbon film and ultraviolet (UV)-visible Shimadzu RF5301PC double beam spectrophotometer. TEM characterization revealed a spherical shape for the nanoparticles with 25 nm in size, and the absorption spectra of the final product were scanned at 200-800 cm^−1^ using 1 cm quartz cells. The spectra of the formed product revealed a transition point at 367 nm with no clear maximum. Meanwhile, commercial inorganic Se was purchased as selenite oily preparation (Na_2_SeO_3_, molecular weight 172.94, 100 mg.L^−1^) from the market (EMIC Veterinary).

Synthesized NS and commercial Se (sodium selenite – EMIC Veterinary) at a rate of 100 mg.L^−1^ were provided to broilers in the drinking water. Treatments were assigned to broiler groups as follow: G1 was supplemented with 0.5 mL NS 100 mg.L^−1^/L producing a final concentration of 0.05 mg.L^−1^, G2 was supplemented with 1.0 mL NS 100 mg.L^−1^/L producing a final concentration of 0.1 mg.L^−1^, G3 was supplemented with 0.5 mL inorganic Se 100 mg.L^−1^/L producing a final concentration of 0.05 mg.L^−1^, G4 was supplemented with 1.0 mL inorganic Se 100 mg.L^−1^/L producing a final concentration of mg.L^−1^, and G5 was kept under untreated control.

### *E. coli* O157: H7 culture propagation and infection

*E. coli* O157: H76.4×10^4^ colony-forming unit culture was purchased from Animal Health Research Institute – Ismailia. The culture was propagated using MacConkey broth and incubated at 44°C/24 h. Drop plate technique was followed, as 10 μL were transferred aseptically from yellow-colored positive MacConkey tubes, dropped onto Eosin Methylene Blue (EMB) agar, and incubated at 37°C/24 h [24]. Typical metallic green colonies were counted and picked up. Four out of the five groups (G1, G2, G3, and G4) were challenged with *E. coli* O157: H7 2.6×10^8^ at the 14^th^ day of age in drinking water [25].

### Performance indices (PI)

Average live body weights (LBW) were determined weekly by weighing 45 birds from each group. The number of weighted birds was calculated using a simple random sampling design [26] with an expected error of 5% using the following formula:

n=1.96^2^ P_exp_ (1−P_exp_)*/*d2

where n = required sample size, P_exp_ = expected prevalence, and d = desired absolute precision. Weekly feed intakes (FI/g) in each group were calculated by dividing the total amount consumed by the number of birds in such a group. Body-weight gains (WG/g), feed conversion ratios (FCR/%), and PI were calculated as recommended by Soliman and Hassan [27].

## Sampling

A total number of 250 birds were sacrificed, including 50 birds per each treatment and control. A total of 2250 samples, including 250 sera, 250 intestinal swabs, 1500 organs, and tissue samples including liver, spleen, heart, bursa, intestine, and breast muscles, and 250 (two sets of 125 samples) eviscerated carcass samples associated with its liver were collected. The blood samples (a total of 250 blood sample for sera separation from 50 birds per each treatment and control) were collected while sacrificing the birds, kept in a water bath at 37°C for 20 min, centrifuged at 3500 rpm for 20 min, and non-hemolyzed sera were piped into Eppendorf tubes and stored at −20°C until used for the immunological assay. Carcasses were weighed after de-feathering and evisceration. The liver, heart, spleen, and bursa of Fabricius were obtained from each carcass, weighed, and expressed as gram per kilogram carcass weight (g/kg). Weighed organs such as liver, spleen, and bursa, and intestines were directed for histopathological and immunohistochemistry examination. One set of 125 eviscerated carcass samples was kept frozen until directed for meat quality examinations, and the second set (125 eviscerated carcasses associated with its liver) was kept frozen for 24 h until directed for RP-HPLC examination for detecting NS and Se tissue residues in correlation to post-sacrificing time factor (1^st^, 5^th^, 10^th^, 15^th^, 20^th^, 25^th^, 30^th^, 35^th^, and 40^th^ days). Intestinal swabs and breast muscle samples were collected on 9 mL buffered peptone water and directed for bacteriological assessment.

### Meat quality assay

Eviscerated carcass samples were examined for drip loss [28] and water holding capacity [29]. The drip loss was assessed at 0 times and after 72 h for breast meat packaged in a polyethylene bag, they were weighed at 0 times (W_0_/g) then stored at 4°C for 72 h. The excess moisture was wiped off, breast samples were re-weighed after 72 h (W_24_/g), and the drip loss was calculated with the following formula:

Drip loss %=100×(W_0_/g−W_24_/g)/W_0_/g

Four replicates of 10 g muscles were sampled, minced, added with 15 mL of 0.6 M sodium chloride solution into 50 mL falcon tubes, and mixed using vortex for 1 min. The tubes were refrigerated at 4°C for 15 min and centrifuged at 4°C by 3000 rpm for 15 min. The water uptake (%) was determined by the formula:

Water holding capacity % = 100×(W_pellet_−W_raw_)/W_raw_

where W_pellet_ was the weight of solid material at the bottom of tubes after centrifugation and W_raw_ was the weight of the chicken meat sample used for analysis.

### HPLC assay

Frozen chicken tissue samples (−20°C) were prepared by partial thawing at room temperature (25°C) for 30 min, minced, and homogenized in the mincer for 1 min. The samples were subjected to extraction process for NS and Se residues according to Fadel *et al*. [30] using injection volume, 50 μL; flow rate, 1.0 mL/min; UV detection at wavelength, 280 nm for Se and 332nm for NS; column temperature, ambient; stop time, 10 min; post time, 1 min; and mobile phase 100% methanol for Se, Methanol: 10 mM citric acid monohydrate (85/15, v/v-2.5 pH) for NS.

The quantification of residues was obtained and calculated from the area under curves extrapolated automatically by the software (Chem Station, France). The presence of NS in tissues was confirmed by a high-resolution transmission electron microscope at the 20^th^ day (Field Emission Scanning Electron Microscope Model Leo Supra 55, Magnification: 20×−900,000×), according to Mühlfeld *et al*. [31].

### Immunological profile

Sera samples were examined for immunoglobulin G (IgG) and immunoglobulin M (IgM) concentrations using ROCHE Elecsys 1010 Immunoassay Analyzer [32].

### Bacteriological assay

Intestinal swabs and breast muscle extract samples (a total of 500 samples, including 250 intestinal swabs and 250 breast muscles previously collected while sacrificing 50 birds per each treatment and control) were prepared, according to the American Public Health Association [33]. The samples were subjected to ten-fold serial dilution up to 10^−7^. Total bacterial count (TBC) and total *Enterobacteriaceae* count (TEC) were conducted using a drop plate technique, as recommended by Kim and Lee [34] and Soliman *et al*. [35]. TBC was performed using standard plate count agar (SPC, SPC Agar, HIMEDIA^®^ M091, 500g) at 37°C for 24-48 h and TEC was conducted using EMB Agar (EMB, Levine Eosin Methylene Blue Agar, MU022, 500g) at 37°Cfor 24-48 h. Plates were counted using the Dark-field colony counter (Reichert™ Dark-filed Quebec™ Colony Counters – Fisher Scientific), as recommended by Murray *et al*. [36].

### Histopathological examination

Specimens of the liver, duodenum, bursa of Fabricius, and spleen were removed from broiler chickens (a total of 1000 samples include 250 liver, 250 duodenum, 250 bursa, and 250 spleen samples were collected while sacrificing 50 birds per each treatment and control), washed with 5% phosphate-buffered saline, and harvested in buffered formalin saline 10% for histopathological examination, specimens were cut into 5-mm thickness sections, put into tissue cassettes, transferred through a series of alcohols with increasing concentrations for dehydration, cleared by transfer into two changes of xylol, embedded in paraffin, cut into 5 μm thickness section, and stained with hematoxylin and eosin (H&E) [37,38]. Histological sections were examined using a Zeiss Axioplan microscope (Carl Zeiss MicroImaging, Thornwood, NY).

### Immunohistochemistry assay

Immunohistochemistry assay was conducted for the detection of nuclear factor kappa B (NF-κB)/p65, in duodenal tissues using a standard immunohistochemical method as recommended by Eissa and Shoman [39] using rabbit anti-mouse phospho-NF-κB p65 antibody (Thermo Fisher Scientific, USA) (1: 50-100) which is used to stain cells with activated NF-κB transcription factor. The biotin-streptavidin systems were used to observe the markers [40]. Diaminobenzidine allows permanent preparation, so, it is used as a chromogen. Hematoxylin was used as a counterstain. Slides were examined using a Zeiss Axioplan microscope (Carl Zeiss MicroImaging, Thornwood, NY). The captured mages were analyzed using Image J 1. 51 p software (10×), (Wayne Rasband, NIH) for NF-κB positive cells.

### Statistical analysis

Statistical analysis was carried out using Statistical Package for the Social Sciences (SPSS version 21.0, IBM Corp., NY, USA) [41]. The data obtained were analyzed statistically using multifactorial analysis of variance (two-tailed ANOVA) to investigate the overall mean prophylactic influence of Se and NS along with the broiler’s age and their interactions. The statistical model was summarized as follow:

Y_ijk_= μ + α_i_ + β_j_ + (αβ)_ij_ + ε_ijk_

where Y_ijk_ was the measurement of dependent variables; μ was overall mean; α_j_ was the fixed effect of the treatments (Se and NS), β_j_ was the fixed effect of the birds’ age, (αβ)_ij_ was the interaction between treatment and broiler age, and ε_ijk_ was the random error. Pearson’s correlation (r) was calculated to determine the correlation coefficient between immunoglobulin concentrations along with total bacterial and *Enterobacteriaceae* counts in intestine and breast muscles. Total bacterial and *Enterobacteriaceae* counts were transformed into logarithm (Log_10_) using Microsoft Excel 2016. The significance was expressed as highly significant at p<0.01, significant at p≤0.05, and non-significant at p>0.05.

## Results

### Performance traits and crude mortality

Broilers were observed during the entire experimental fattening cycle for the mortalities. The calculated crude mortality rate revealed a total of eight birds (3.07%) concerning the total understudy population size. WG overall means in Table-1 revealed highly significant increases (p<0.01) in broilers supplemented with 0.5 mL NS compared to all other groups with no significant differences between broilers supplemented with 0.5 mL inorganic Se, 1.0 mL NS, and control group.

**Table-1:** Performance indices (Mean±SE) of Hubbard broiler supplemented with different concentrations of selenium and nano-selenium.

Groups	Age/week	WG/g	FI/g	FCR (%)	PI

Overall means in broiler groups					
G1		325.2^a^±5.92	616.7^c^±0.00	1.8^e^±0.03	4.4^a^±0.07
G2		300.0^b^±5.21	622.4^b^±0.00	1.9^d^±0.02	3.7^b^±0.05
G3		295.9^b^±3.36	654.0^a^±0.00	2.1^b^±0.02	3.3^c^±0.08
G4		279.7^c^±2.28	659.4^a^±0.00	2.3^a^±0.01	2.9^d^±0.04
G5		297.2^b^±4.73	626.7^b^±0.00	2.0^c^±0.03	3.5^bc^±0.08
p value		0.000	0.000	0.000	0.000

**Interactions between groups by age of birds**					

G1	1^st^	76.6^d^±3.05	105.5^e^±0.01	1.3^c^±0.05	0.8^e^±0.05
	2^nd^	258.2^c^±3.71	336.1^d^±0.00	1.3^c^±0.01	2.9^d^±0.06
	3^rd^	516.8^a^±5.62	892.1^a^±0.00	1.7^b^±0.01	5.1^c^±0.07
	4^th^	378.5^b^±10.70	861.1^c^±0.00	2.2^a^±0.07	5.6^b^±0.19
	5^th^	396.0^b^±15.96	888.6^b^±0.01	2.2^a^±0.09	7.4^a^±0.35
G2	1^st^	56.0^e^±2.08	109.2^e^±0.00	1.9^b^±0.07	0.5^d^±0.03
	2^nd^	195.1^d^±3.25	345.2^d^±0.01	1.7^b^±0.02	1.6^c^±0.04
	3^rd^	492.0^a^±20.64	902.1^b^±0.01	1.8^b^±0.08	4.3^b^±0.28
	4^th^	347.9^c^±23.98	895.4^c^±0.00	2.6^a^±0.16	4.4^b^±0.34
	5^th^	388.6^b^±22.99	1018.2^a^±0.00	2.7^a^±0.16	5.8^a^±0.41
G3	1^st^	76.9^d^±1.12	106.1^d^±0.00	1.3^c^±0.02	0.8^d^±0.02
	2^nd^	228.7^c^±7.97	325.1^c^±0.01	1.4^c^±0.05	2.4^c^±0.13
	3^rd^	507.0^a^±11.59	902.1^a^±0.01	1.7^b^±0.04	4.8^b^±0.14
	4^th^	337.5^b^±7.43	888.8^b^±0.01	2.6^a^±0.06	4.5^b^±0.10
	5^th^	350.2^b^±9.67	890.1^b^±0.00	2.5^a^±0.07	6.0^a^±0.20
G4	1^st^	50.6^e^±0.54	112.3^d^±0.00	2.2^b^±0.02	0.4^d^±0.01
	2^nd^	166.0^d^±2.84	354.1^c^±0.01	2.1^b^±0.03	1.2^c^±0.03
	3^rd^	461.8^a^±6.20	906.1^b^±0.02	1.9^c^±0.02	3.6^b^±0.07
	4^th^	385.4^b^±8.23	902.4^b^±0.01	2.3^b^±0.05	4.7^a^±0.11
	5^th^	334.8^c^±9.90	1022.1^a^±0.01	3.0^a^±0.08	4.7^a^±0.17
G5	1^st^	58.0^d^±5.47	109.1^d^±0.01	1.8^b^±0.05	0.5^d^±0.02
	2^nd^	211.1^c^±9.12	331.1^c^±0.00	1.5^c^±0.05	2.0^c^±0.14
	3^rd^	504.6^a^±8.57	914.2^a^±0.00	1.8^b^±0.03	4.5^b^±0.09
	4^th^	360.4^b^±4.42	887.1^b^±0.00	2.4^a^±0.03	4.7^b^±0.06
	5^th^	352.1^b^±9.62	892.1^b^±0.00	2.5^a^±0.07	6.0^a^±0.19
p value		0.000	0.000	0.000	0.000

Means carrying different superscripts in the same column are significantly different at (p≤0.05) or highly significantly different at (p<0.01). Means carrying the same superscripts in the same column are non-significantly different at p*>*0.05. G1=Hubbard with 0.5 mL nano-selenium/1 L drinking water, G2=Hubbard with 1.0 mL nano-selenium/1 L drinking water, G3=Hubbard with 0.5 mL selenium/1 L drinking water), G4=Hubbard with 1.0 mL selenium/1 L drinking water, and G5=control. WG=Weight gain, FI=Feed intake, FCR=Feed conversion ratio, and PI=Performance index, SE=Standard error

Feed intakes overall mean (Table-1) revealed highly significant increases (p<0.01) in broilers supplemented with 0.5 mL and 1.0 mL inorganic Se with no significant differences between the two groups compared to broilers supplemented with NS and to control broilers.

FCR, as shown in Table-1, revealed highly significant improvement (p<0.01) in broilers supplemented with 0.5 mL NS, 1.0 mL NS, control, 0.5 mL inorganic Se, and 1.0 mL inorganic Se, respectively. The calculated improvement of FCR in G1 and G2 broilers supplemented with NS (0.5 and 1.0 mL) and in the G5 control group might be attributed to the lower feed intake (numerator) and higher WG (denominator) compared to G3 and G4 broilers supplemented with inorganic Se (sodium selenite, EMIC Veterinary).

Overall means of PI in Table-1 revealed highly significant increases (p<0.01) in broilers supplemented with 0.5 mL NS, 1.0 mL NS, control, 0.5 mL inorganic Se, and 1.0 mL inorganic Se, respectively, with no significant differences between broilers supplemented with 1.0 mL NS and 0.5 mL Se with the control group. The calculated improvement of performance index in G1 and G2 broilers supplemented with NS (0.5 and 1.0 mL) and in the G5 control group might be attributed to the higher body weight per kg (numerator) and higher FCR (denominator) compared to G3 and G5 broilers supplemented with inorganic Se (sodium selenite, EMIC Veterinary).

### Live body, carcass, and organs weight

LBW, as revealed in Table-2, recorded highly significant increases (p<0.01) in broilers supplemented with 0.5 mL NS, 1.0 mL NS, 0.5 mL inorganic Se, 1.0 mL inorganic Se, and control, respectively, with no significant differences between LBW of broilers supplemented with 1.0 mL inorganic Se and control untreated broilers. Carcasses’ weights revealed highly significant increases (p<0.01) in broilers supplemented with 0.5 mL NS, 1.0 mL NS, 0.5 mL inorganic Se, 1.0 mL inorganic Se, and control with no significant differences between carcass weight values in broilers supplemented with 0.5 inorganic Se and 1.0 mL NS.

**Table-2:** Carcass quality (Mean±SE) and organs percentage (Mean±SE) of Hubbard broiler supplemented with different concentrations of selenium and nano-selenium.

Groups	LBW/g	Carcass weight/g	Immune and edible organs weights/g			

			Liver	Spleen	Heart	Bursa
**Overall means in broiler groups**						

G1	2294^a^±21.6	2016^a^±15.8	50.4^a^±0.20	3.0^a^±0.015	12.9^a^±0.03	1.6^a^±0.00
G2	1994^b^±21.6	1761^b^±15.8	44.6^b^±0.20	2.4^b^±0.015	10.4^b^±0.03	1.3^b^±0.01
G3	1859^c^±25.3	1741^b^±18.6	40.7^c^±0.23	2.3^b^±0.018	9.7^c^±0.04	1.1^c^±0.01
G4	1778^d^±21.6	1538^c^±15.8	40.4^c^±0.20	2.0^c^±0.015	9.0^d^±0.03	0.9^d^±0.01
G5	1731^d^±25.3	1359^d^±18.6	23.1^d^±0.23	1.3^d^±0.018	5.6^e^±0.04	0.6^e^±0.01
p value	0.000	0.000	0.041	0.033	0.020	0.012

Means carrying different superscripts in the same column are significantly different at (p≤0.05) or highly significantly different at (p<0.01). Means carrying the same superscripts in the same column are non-significantly different at (p>0.05). G1=Hubbard with 0.5 mL nano-selenium/1 L drinking water, G2=Hubbard with 1.0 mL nano-selenium/1 L drinking water, G3=Hubbard with 0.5 mL selenium/1 L drinking water), G4=Hubbard with 1.0 mL selenium/1 L drinking water, and G5=Control. LBW=Live body weight, SE=Standard error

The liver weights revealed in Table-2 highly significant increases (p<0.01) in broilers supplemented with 0.5 mL NS, 1.0 mL NS, 0.5 mL inorganic Se, and 1.0 mL inorganic Se, respectively, compared to control group with no significant differences between liver weights from broilers supplemented with 0.5 and 1.0 mL inorganic Se (sodium selenite, EMIC Veterinary). The spleen weights revealed highly significant increases in broilers supplemented with 0.5 mL NS, 1.0 mL NS, 0.5 mL Se, and 1.0 mL Se, respectively, compared to the control group with no significant differences between spleen weights from broilers supplemented with 1.0 mL NS and 0.5 mL Se (Table-2). Heart and bursa of Fabricius weights (Table-2) revealed highly significant increases (p<0.01) in broilers supplemented with 0.5 mL NS, 1.0 mL NS, 0.5 mL inorganic Se, and 1.0 mL inorganic Se, respectively, compared to control group.

### Meat quality parameters

Initial pH_0_ values revealed highly significant declines (p<0.01), as shown in Table-3, in control and broilers supplemented with 0.5 mL inorganic Se with no significant differences between the values in broilers supplemented with 0.5 mL NS, 1.0 mL NS, and 1.0 mL inorganic Se. Meanwhile, pH_24_ values revealed highly significant declines (p<0.01) in broilers supplemented with 0.5 mL NS compared to control and other supplemented groups.

**Table-3:** Meat quality parameters (Mean±SE) in Hubbard broilers supplemented with different concentrations of selenium and nano-selenium.

Groups	pH_ 0_	pH_ 24_	Drip loss %	Water holding capacity %
**Overall means in broiler groups**				

G1	7.21^a^±0.059	5.56^c^±0.144	1.54^c^±0.219	9.60^a^±1.281
G2	7.25^a^±0.021	6.08^b^±0.035	2.68^bc^±0.397	7.70^a^±0.854
G3	6.98^b^±0.103	6.47^a^±0.185	3.33^b^±0.200	8.10^a^±0.680
G4	7.21^a^±0.012	6.18^ab^±0.016	3.92^ab^±0.131	8.85^a^±0.427
G5	6.58^c^±0.046	5.73^c^±0.063	4.89^a^±0.931	5.80^a^±0.081
p value	0.000	0.000	0.002	0.236

Means carrying different superscripts in the same column are significantly different at (p≤0.05) or highly significantly different at p*<*0.01. Means carrying the same superscripts in the same column are non-significantly different at p*>*0.05. G1=Hubbard with 0.5 mL nano-selenium/1 L drinking water, G2=Hubbard with 1.0 mL nano-selenium/1 L drinking water, G3=Hubbard with 0.5 mL selenium/1 L drinking water), G4=Hubbard with 1.0 mL selenium/1 L drinking water, and G5=Control. pH_ 0_=Hydrogen ion concentrations at 0 time, pH_ 24_=Hydrogen ion concentrations 24 h

Drip loss (Table-3) revealed highly significant declines in broilers supplemented with 0.5 and 1.0 mL NS with no significant differences between the two groups. Meanwhile, water holding capacity (Table-3) revealed no significant differences between all supplemented groups and control.

### Immunoglobulin concentrations and bacterial loads

IgG and IgM concentrations, in Table-4, revealed highly significant increases (p<0.01) in broilers supplemented with 0.5 mL NS, 1.0 mL NS, 1.0 mL inorganic Se, 0.5 mL inorganic Se, and control group, respectively, with no significant differences in IgG concentrations between broilers supplemented with 0.5 and 1.0 mL inorganic Se.

**Table-4:** Immunoglobulin concentration and logarithm bacterial load (Mean±SE) of Hubbard broiler supplemented with different concentrations of selenium and nano-selenium.

Groups	Immunoglobulin concentration		Bacterial counts			
	
	IgG (mg/dl)	IgM (mg/dl)	Intestinal swabs		Breast muscles	
	
			Log. TBC (CFU/mL)	Log. TEC (CFU/mL)	Log. TBC (CFU/mL)	Log. TEC (CFU/mL)
**Overall means in broiler groups**						

G1	1710.3^a^±2.3	390.1^a^±1.8	5.39^d^±0.018	3.99^e^±0.013	3.64^d^±0.015	1.64^e^±0.017
G2	1680.0^b^±5.7	373.6^b^±1.6	5.80^c^±0.016	4.22^c^±0.012	4.23^c^±0.014	2.10^c^±0.016
G3	1619.1^c^±6.9	333.5^d^±2.1	5.93^b^±0.021	4.35^b^±0.015	4.51^b^±0.018	2.39^b^±0.020
G4	1626.6^c^±4.7	354.4^c^±1.8	6.54^a^±0.018	4.48^a^±0.013	4.57^a^±0.015	2.44^a^±0.017
G5	1366.0^d^±8.8	274.2^e^±2.1	3.88^e^±0.021	4.05^d^±0.015	2.77^e^±0.018	1.74^d^±0.020
p value	0.000	0.001	0.000	0.000	0.000	0.000

Means carrying different superscripts in the same column are significantly different at (p≤0.05) or highly significantly different at (p<0.01). Means carrying the same superscripts in the same column are non-significantly different at (p>0.05). G1=Hubbard with 0.5 mL nano-selenium/1 L drinking water, G2=Hubbard with 1.0 mL nano-selenium/1 L drinking water, G3=Hubbard with 0.5 mL selenium/1 L drinking water), G4=Hubbard with 1.0 mL selenium/1 L drinking water, and G5=Control. IgG=Immunoglobulin G, IgM=Immunoglobulin M, TBC=Total Bacterial count, TEC=Total *Enterobacteriaceae* count, CFU=Colony-forming unit, SE=Standard error

A synchronized highly significant declines (p<0.01), as shown in Table-4, in total bacterial and *Enterobacteriaceae* counts of intestinal swabs and breast muscles in broilers supplemented with 0.5 and 1.0 mL NS compared to other supplemented groups, with high significant declines (p<0.01) in broilers supplemented with 0.5 mL NS compared to those supplemented with 1.0 mL NS.

Pearson’s correlation in Table-5 indicated highly significant positive strong correlations (r=0.714; 0.637, p<0.01) between IgG with the TBC of intestinal swabs and breast muscles, respectively. Meanwhile, IgM revealed highly significant positive strong and medium correlations (r=0.619; 0.504, p<0.01) with the TBC of intestinal swabs and breast muscles, respectively.

**Table-5:** Correlation coefficient between bacterial load in the intestine and/or breast muscles with IgG concentration (above diagonal) and IgM concentration (below diagonal) of Hubbard broiler supplemented with different concentration of selenium and nano-selenium.

r	IgG	TBCI	TBCB	TECI	TECB
IgM	1	0.714**	0.637**	0.134	0.148
TBCI	0.619**	1	0.960**	0.750**	0.745**
TBCB	0.504**	0.960**	1	0.813**	0.833**
TECI	−0.005	0.750**	0.813**	1	0.950**
TECB	−0.013	0.745**	0.833**	0.950**	1

** Correlation is significant at the 0.01 level.

* Correlation is significant at the 0.05 level. ^NS^. Correlation is non-significant at the 0.05 level. IgG=Immunoglobulin IgG, IgM=Immunoglobulin IgM, TBCI=Total Bacterial Count in the intestine, TBCB=Total Bacterial Count in Breast Muscle, TECI=Total *Enterobacteriaceae* Count in the intestine, TECB=Total *Enterobacteriaceae* Count in breast muscle, r=Pearson’s correlation coefficient

### Se and NS tissue residues

**Figure-1:**
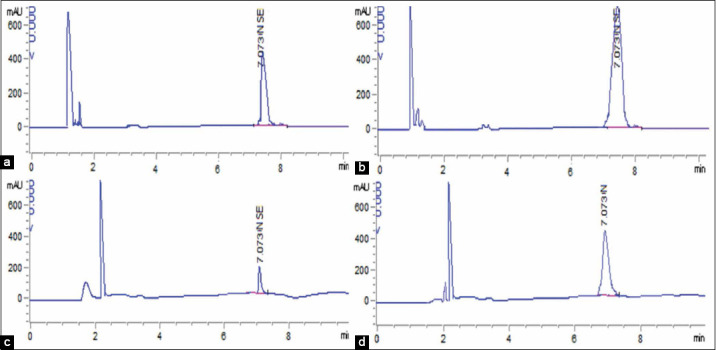
Liquid chromatography of nano-selenium residues in liver and muscles at 1st day following supplementation with: (a) 0.5 mL of 100 mg.L^−1^ in the liver, (b) 1.0 mL of 100 mg.L^−1^ in the liver, (c) 0.5 mL of 100 mg.L^−1^ in muscles. (d) 1.0 mL of 100 mg.L^−1^ in muscles.

**Figure-2:**
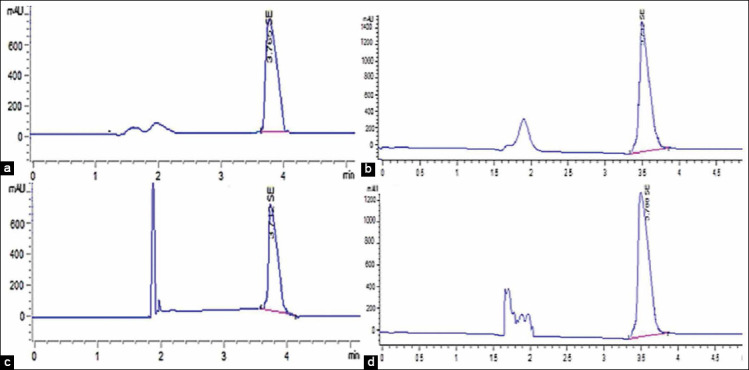
Liquid chromatography of selenium residues in liver and muscles at 1st day following supplementation with: (a) 0.5 mL of 100 mg.L^−1^ in the liver, (b) 1.0 mL of 100 mg.L^−1^ in the liver, (c) 0.5 mL of 100 mg.L^−1^ in muscles. (d) 1.0 mL of 100 mg.L^−1^ in muscles.

**Figure-3:**
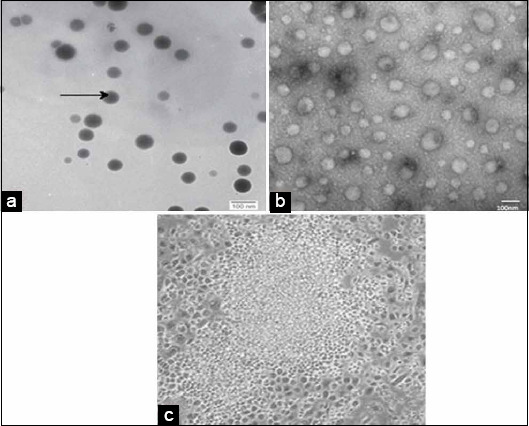
Scanning electron microscopy images of nano-selenium (NS) in liver samples on the 20th day. (a) The liver of broilers supplemented with 1.0 mL NS (0.1 mg.L^−1^) on the 20th day showed NS particles (arrow) of 23.8 nm in size and 0.0528 µg/g in concentration. (b) The liver of broilers supplemented with 0.5 mL NS (0.05 mg.L^−1^) on the 20th day confirmed the disappearance of NS particles. (c) The liver of un-supplemented control broilers on the 20th day. Scale bar: 100 nm.

The quantification of NS and Se residues in the liver and muscles of broilers in Table-6 showed highly significant declines (p≤0.01) of NS residues in broilers supplemented with 0.5 and 1.0 mL NS compared to Se residues in broilers supplemented with 0.5 and 1.0 mL inorganic Se. Highly significant increases (p<0.001) in the concentration of NS and Se residues as revealed in Figures-1 and 2 were detected in the liver compared to muscles. Electron microscopy images that were taken on the 20^th^ day as shown in Figure-3, revealed the presence of NS particles in the liver of broiler supplemented with 1 mL NS (0.1 mg.L^−1^, Figure-3a) and its disappearance from the liver of broilers supplemented with 0.5 mL NS (0.05 mg.L^−1^, Figure-3b) compared to control (Figure-3c). Inorganic Se (0.5 and 1.0 mL) was detectable in both liver and muscles up to 45^th^-day samples, 0.5 mL NS disappeared in muscles at the 20^th^ day and in liver at the 30^th^-day samples, and 1.0 mL NS disappeared in muscles at 30^th^ day and in liver at 35^th^-day samples. Thus, NS residues disappeared earlier than inorganic Se from broiler tissues (liver and muscles). The results of the examination by a high-resolution transmission electron microscope confirmed the presence of NS in the liver sample of G1 on the 20^th^ day and disappeared on the 30^th^ day.

**Table-6:** Selenium and nano-selenium tissue residues (Mean±SE) in the liver and muscle of Hubbard broilers using RP-HPLC.

Groups	Age/days	Tissue residues (Mean±SE) mg/g	

		Muscles	Liver
G1		0.158^d^±0.006	0.221^d^±0.010
G2		0.427^b^±0.003	0.716^b^±0.011
G3		0.338^c^±0.002	0.448^c^±0.008
G4		0.830^a^±0.002	1.408^a^±0.010
p value		0.000	0.000

Selenium*age			

G1	1^st^	0.8780^a^±0.039	1.0193^a^±0.006
	5^th^	0.4504^b^±0.016	0.6057^b^±0.034
	10^th^	0.1620^c^±0.029	0.3699^c^±0.020
	15^th^	0.0847^d^±0.005	0.1591^d^±0.022
	20^th^	ND	0.0562^e^±0.005
	25^th^	ND	ND
	30^th^	ND	ND
	35^th^	ND	ND
	40^th^	ND	ND
	45^th^	ND	ND
G2	1^st^	1.8375^a^±0.062	2.0409^a^±0.030
	5^th^	0.9208^b^±0.034	1.1663^b^±0.075
	10^th^	0.4207^c^±0.012	0.6756^c^±0.024
	15^th^	0.1448^d^±0.016	0.3311^d^±0.013
	20^th^	0.0571^e^±0.004	0.1550^e^±0.011
	25^th^	ND	0.0790^f^±0.003
	30^th^	ND	0.0323^g^±0.006
	35^th^	ND	ND
	40^th^	ND	ND
	45^th^	ND	ND
G3	1^st^	1.0683^a^±0.026	1.9262^a^±0.052
	5^th^	0.9109^b^±0.002	1.5568^b^±0.054
	10^th^	0.7427^c^±0.025	1.0897^c^±0.020
	15^th^	0.5249^d^±0.009	0.9604^c^±0.022
	20^th^	0.4058^e^±0.003	0.6390^d^±0.049
	25^th^	0.3238^f^±0.006	0.5191^e^±0.014
	30^th^	0.1797^g^±0.012	0.2281^f^±0.063
	35^th^	0.0918^h^±0.003	0.1059^g^±0.007
	40^th^	0.0182^i^±0.004	0.0873^h^±0.003
	45^th^	ND	0.0448^i^±0.001
G4	1^st^	2.0827^a^±0.039	2.9271^a^±0.037
	5^th^	1.5024^b^±0.010	2.3936^b^±0.064
	10^th^	1.2930^c^±0.021	2.0509^c^±0.040
	15^th^	1.0913^d^±0.020	1.7981^d^±0.017
	20^th^	0.8077^e^±0.007	1.1194^e^±0.065
	25^th^	0.6332^f^±0.005	1.1072^f^±0.003
	30^th^	0.4805^g^±0.025	0.9718^g^±0.025
	35^th^	0.2486^h^±0.032	0.8426^h^±0.034
	40^th^	0.1197^i^±0.010	0.6441^i^±0.032
	45^th^	0.0367^j^±0.005	0.2219^j^±0.011
p value		0.000	0.000

Means carrying different superscripts in the same column are significantly different at p*≤*0.05 or highly significantly different at p*<*0.01. Means carrying the same superscripts in the same column are non-significantly different at p*>*0.05. G1=Hubbard with 0.5 mL nano-selenium/1 L drinking water, G2=Hubbard with 1.0 mL nano-selenium/1 L drinking water, G3=Hubbard with 0.5 mL selenium/1 L drinking water, G4=Hubbard with 1.0 mL selenium/1 L drinking water, and G5=Control. ND=Not detected, SE=Standard error

### Tissue architecture

**Figure-4:**
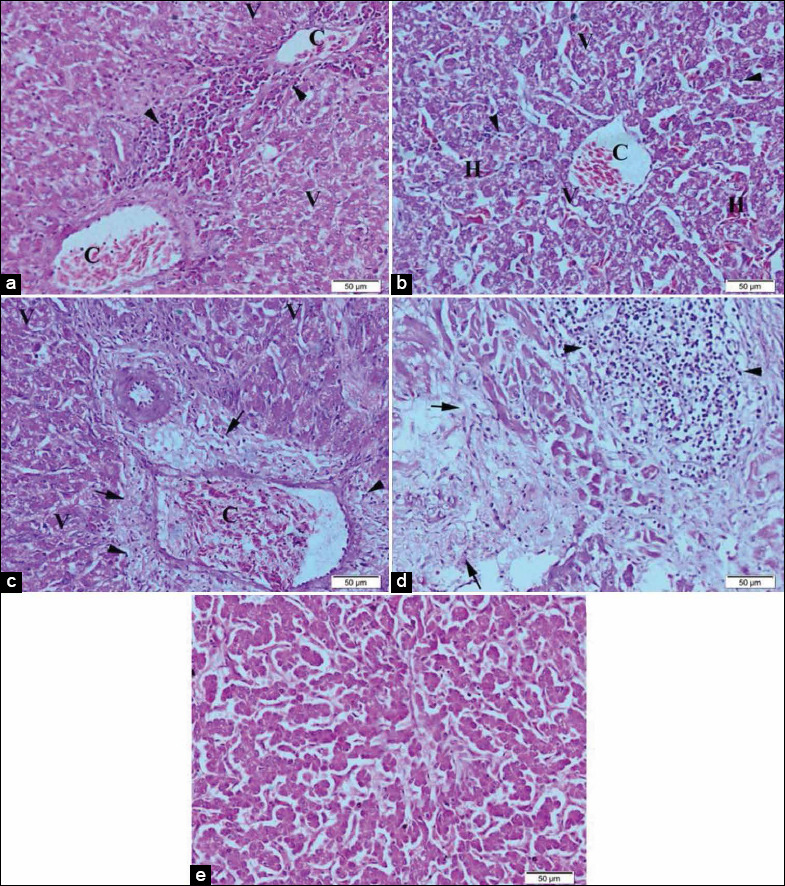
Representative photomicrographs of liver histopathology (20×): (a) The liver of Hubbard chicken after oral administration of 0.5 mL nano-selenium (NS) showing congestion of blood vessels (C), mononuclear cell infiltration (arrowhead), vaculation of hepatocytes cytoplasm (V). (b) The liver of Hubbard chicken after oral administration of 1.0 mL NS showing congestion of central vein, hemorrhage (H), and vaculation of hepatocytes(V). (c) Liver of Hubbard chicken after oral administration of 0.5 mL selenium (Se) showing congestion of portal blood vessels (C), mononuclear cell infiltration (arrowhead), vaculation of hepatocytes cytoplasm (V), and fibrosis (arrow). (d) Liver of Hubbard chicken after oral administration of 1.0 mL Se. (e) The liver of control Hubbard. Hematoxylin and eosin. Bar 50 μm.

Liver photomicrographs revealed in Figure-4a congestion of central vein and portal blood vessels, hepatic cells showed degeneration with mild vaculation of cytoplasm and moderate mononuclear cell infiltration in broilers supplemented with 0.5 mL NS. The liver of broilers supplemented with 1.0 mL NS (Figure-4b) showed congestion of central vein and hepatic cells showed degeneration with severe vaculation of cytoplasm and mononuclear cell infiltration, with multiple areas of hemorrhage. Broilers supplemented with 0.5 mL inorganic Se showed in Figure-4c congestion of portal blood vessels, severe biliary cirrhosis, while hepatocytes showed moderate degeneration, cytoplasmic vaculation, and mononuclear cell infiltration. The liver of broilers supplemented with 1.0 mL inorganic Se showed in Figure-4d severe degeneration of hepatocytes, severe intrahepatic fibrosis, and severe mononuclear cell infiltration compared to normal architecture in Figure-4e.

**Figure-5:**
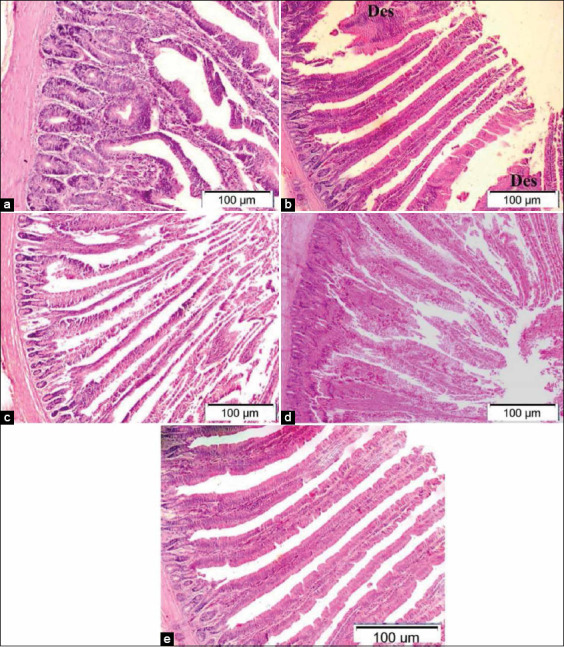
Representative photomicrographs of duodenum histopathology (10×): (a) Duodenum of Hubbard chicken after oral administration of 0.5 mL nano-selenium (NS). (b) Duodenum of Hubbard chicken after oral administration of 1.0 mL NS showing mild desquamation of villi (des). (c) Duodenum of Hubbard chicken after oral administration of 0.5 mL selenium (Se). (d) Duodenum of Hubbard chicken after oral administration of 1.0 mL Se. (e) Duodenum of control Hubbard. Hematoxylin and eosin. Bar 100 μm.

Intestinal histopathological examination of broilers supplemented with 0.5 mL NS revealed in Figure-5a maintenance of intestinal villi, glands, and muscular layer with mild destruction of lining epithelium of villi compared to normal view in Figure-5e. Broilers supplemented with 1.0 mL NS showed in Figure-5b maintained intestinal villi with increased length of villi, other areas showed degeneration of villi with an increased number of goblet cells. Figure-5c revealed severe degeneration of intestinal villi with a maintained glandular and muscular layer in broilers supplemented with 0.5 mL inorganic Se. The intestine of broilers supplemented with 1.0 mL inorganic Se revealed (Figure-5d) severe degenerations of intestinal villi remnant was present, with maintained glandular and muscular layer compared to normal view in Figure-4e.

**Figure-6:**
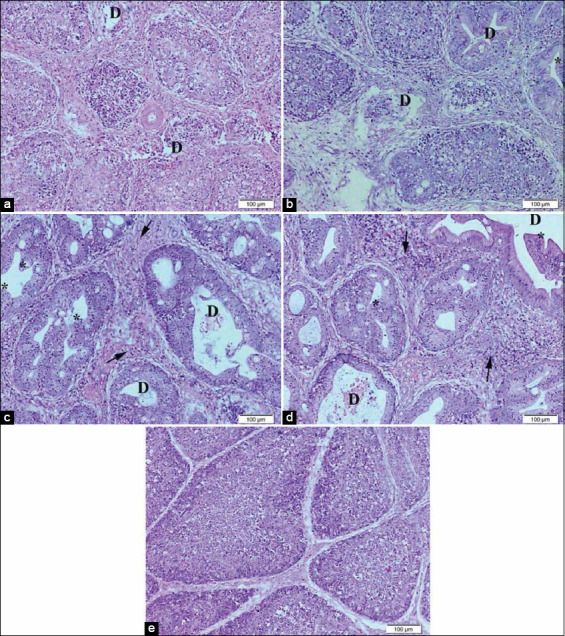
Representative photomicrographs of bursa histopathology (10×): (a) Bursa of Hubbard chicken supplemented with 0.5 mL nano-selenium (NS) showing mild depletion of lymphocytes (D). (b) Bursa of Hubbard chicken supplemented with 1.0 mL NS showing mild depletion of lymphocytes (D) and hyperplasia of follicular epithelium (*). (c) Bursa of Hubbard chicken supplemented with 0.5 mL selenium (Se) showing severe depletion of lymphocytes (D) hyperplasia of follicular epithelium (*) and increased interfollicular fibrosis (arrow). (d) Bursa of Hubbard chicken supplemented with 1.0 mL Se. (e) Bursa of control Hubbard. Hematoxylin and eosin. Bar 100 μm.

Photomicrographs of the bursa of Fabricius in comparison to normal view in Figure-6e showed mild depletion of lymphoid follicles in broilers supplemented with 0.5 mL NS (Figure-6a), hyperplasia of follicular epithelium and moderate lymphoid depletion in the lymphoid follicles of broilers supplemented with 1.0 mL NS (Figure-6b), severe hyperplasia of follicular epithelium and severe depletion of lymphocytes which was replaced by an edematous fluid with increased interfollicular fibrosis in broilers supplemented with 0.5 mL inorganic Se (Figure-6c), and severe interfollicular fibrosis, severe depletion of lymphoid follicles, and hyperplasia of follicles epithelium in broilers supplemented with 1.0 mL inorganic Se (Figure-6d).

**Figure-7:**
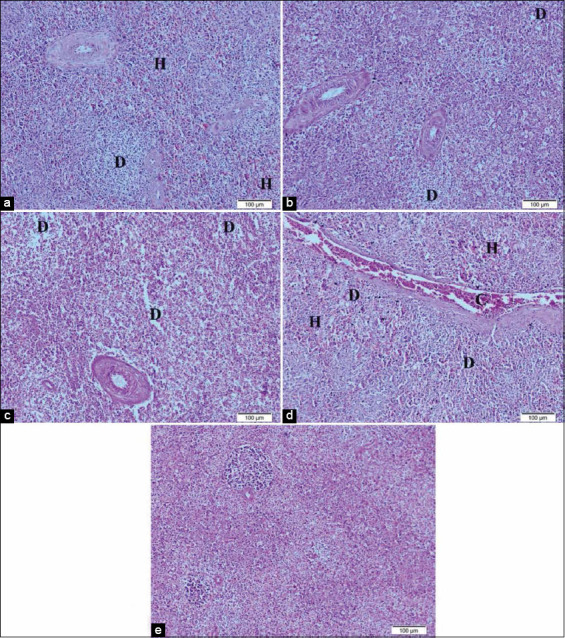
Representative photomicrographs of spleen histopathology (10×): (a) Spleen of Hubbard chicken after oral administration of 0.5 mL nano-selenium (NS) showing depletion of lymphocytes (D) and mild hemorrhage (H). (b) The spleen of Hubbard chicken after oral administration of 1.0 mL NS. (c) The spleen of Hubbard chicken after oral administration of 0.5 mL selenium (Se). (d) The spleen of Hubbard chicken after oral administration of 1.0 mL Se showing depletion of lymphocytes (D), hemorrhage, and severe congestion of splenic sinus (C). (e) The spleen of control Hubbard chicken Hematoxylin and eosin. Bar 100 μm.

Spleen histopathological photomicrographs in broilers supplemented with 0.5 mL NS (Figure-7a) showed mild lymphoid depletion with a moderate area of hemorrhage, in broilers supplemented with 1.0 mL NS (Figure-7b) they revealed moderate lymphoid depletion, in broilers supplemented with 0.5 mL inorganic Se (Figure-7c) they showed severe lymphoid depletion, and in broilers supplemented with 1.0 mL inorganic Se (Figure-7d) they showed an area of hemorrhage with hemosiderosis, severe congestion of splenic sinus and mild lymphoid depletion compared to normal splenic view in Figure-7e.

**Figure-8:**
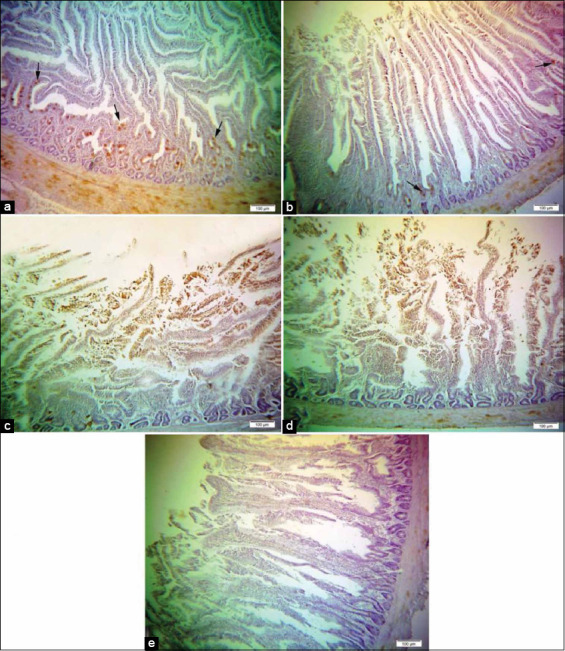
Immunohistochemical detection of phospho-nuclear factor kappa B (NF-κB) p65 in the duodenum of Hubbard chicken; (a) duodenum of Hubbard chicken after oral administration of 0.5 mL nano-selenium (NS). (b) Duodenum of Hubbard chicken after oral administration of 1.0 mL NS. (c) Duodenum of Hubbard chicken after oral administration of 0.5 mL selenium (Se). (d) Duodenum of Hubbard chicken after oral administration of 1.0 mL Se. (e) Duodenum of control Hubbard. Arrows refer to phospho-NF-κB p65 expression, which positively stained brown in the nuclei of glands and epithelial cells. (10×) Bar 100 μm.

Tissue sections of the duodenum from control Hubbard chicken demonstrated low expression of NF-kB p65 (Figure-8e), meanwhile, tissue sections of the duodenum from Hubbard broilers supplemented with 0.5 mL and 1.0 mL inorganic Se shown in Figure-8a and b, revealed overexpression of NF-kB p65, particularly in the nuclei of glands and epithelial cells. In contrast, the section of the duodenum from Hubbard broilers supplemented with 0.5 mL and 1.0 mL NS shown in Figure-8c and d, revealed significantly low expression of NF-kB p65 staining.

## Discussion

Se is a trace element known for its important role in biological processes inside the body and in enhancing bird’s performance and growth, as reported by Lee *et al*. [42] and Limaye *et al*. [43]. Kieliszek and Błażejak [44] stated the importance of Se as micronutrients, as it helps in the protection from hydrogen peroxide, detoxification of heavy metals, enhancement of production and performance, and improvement of the immune system. Kumaran *et al*. [45] reported that NS revealed better improvements in growth performance compared to sodium selenite when used in the ration of Vencobb broilers. Liu *et al*. [46] recorded that supplementing broilers with 0.3 ppm NS improved growth and performance over Se. Ahmadi *et al*. [47] reported improved performance, immunity, and carcass characteristics without any pathological alternations in organs and tissues of broilers supplemented with 0.2, 0.3, 0.4, and 0.5 ppm of NS.

Earlier studies recommended the usage of 0.5 mL and/or 1.0 mL NS or inorganic Se (100 mg.L^−1^) water supplementation in the broiler industry. Hassan *et al*. [12] reported significant improvements by the usage of 0.5 mL NS 100 mg.L^−1^ (0.05 mg.L^−1^) over inorganic Se (sodium selenite) on growth traits, antioxidant activity, immunoglobulin concentration, biochemical profile, behavioral patterns, and histopathological pictures in two experimented broiler breeds (Arbor and Ross) exposed to heat stress. Meanwhile, Ali *et al*. [23] recommended the usage of 1 mL NS 100 mg.L^−1^ (0.1 mg.L^−1^) and reported significant improvements of performance, behavioral patterns, antioxidant activity, immunoglobulin concentration, biochemical profile, and histopathological pictures in two experimented broiler breeds (Arbor Acres^®^ and Ross^®^ 308) exposed to the microbial challenge when comparing its influence to inorganic Se (sodium selenite). That is why the current study focused on conducting a comparison on the effect of different doses (0.5 and 1.0 mL at a rate of 100 mg.L^−1^) of NS and inorganic Se (sodium selenite) on different parameters including growth traits, behavior, carcass characteristics, immunoglobulin concentration, intestinal bacterial load, and photomicrographs of some edible and immune organs in broiler chickens to give leverage to one of the examined doses.

The examinations and evaluations, in our study, revealed significant improvements in broilers supplemented with 0.5 mL NS at a rate of 100 mg.L^−1^ (0.05 mg.L^−1^) compared to those supplemented with 1.0 mL NS at a rate of 100 mg.L^−1^ (0.1 mg.L^−1^) in all tested aspects. The superior influences recorded in using 0.5 mL NS at a rate of 100 mg.L^−1^ (0.05 mg.L^−1^) might be attributed to the smaller size of particles that enhanced their preventive influence. Benko *et al*. [48] examined the influence and toxicity of organic Se, inorganic Se, and NS on histological architecture and they revealed that NS (100-500 nm) was more toxic compared to organic Se (Sel-Plex and Lact-Micro Se) and inorganic Se (selenate more toxic than hydroselenite). They related the toxicity to the particle size diameter of the compound used, as larger compounds tend to be more toxic than smaller. Furthermore, Peng *et al*. [49] suggested using NS in a particle size diameter smaller than 100 nm to produce higher chemopreventive influence.

The current study revealed significant improvements in the productive performance of broilers supplemented with 0.5 mL rather than 1.0 mL NS (100 mg.L^−1^) per liter drinking water, and the results were supported by those of Saleh [50] who recorded an increase in growth performance and attributed this increase to the protein digestibility and energy utilization. Marković *et al*. [51] reported an enhancement in productive performance, health, immunity, egg, and meat quality in broilers under physiological stresses and supplemented with NS. Shabani *et al*. [52], in agreement with our results, revealed that using 200 μg Se daily in broilers can enhance body WG. Zhao *et al*. [53] supplemented broiler breeders with 0.15 or 0.30 mg/kg Se and revealed an improvement in WG. On the contrary, the results did not agree with those of Hassan *et al*. [54] who used 0.3 mg Se /kg for supplementing 360 50-day-old male chickens and found no significant changes in feed intakes and body WG, but there were significant improvements in antioxidant activity and carcass meat quality in these chickens.

The results also revealed significant improvements in carcass and meat quality, significant reductions in pH_24_, and non-significant reductions in both drip loss and water holding capacity in broilers supplemented with 0.5 mL.L^−1^ drinking water NS in agreement with Visha *et al*. [55] who supplemented broilers with 0.3 mg sodium selenite/kg, 0.3 mg organic Se/kg, and NS at three levels (0.15, 0.3, and 0.6 mg/kg). They revealed that broilers supplemented with NS showed a significant reduction in breast muscle drip losses and lipid peroxidation. They recommended a dose of 0.3-0.6 mg NS/kg ration for broilers to minimize drip losses and lipid peroxidation to enhance meat quality. Our results were in agreement with those of Yang *et al*. [56] who recorded that supplementary Se different forms did not influence the pH values of breast meat. In agreement with our results, Surai *et al*. [57] recorded a lower drip loss in broilers supplemented with 0.3-0.6 mg/kg NS. Li *et al*. [58] reported as well that broilers supplemented with 0.15 mg/kg NS had a significantly lower drip loss in the breast muscle at 24 and 48 h after slaughter.

Supplementing Hubbard broilers in the current study with 0.5 and 1.0 mL NS were able to significantly improve IgG and M concentrations with a higher degree of increase in broilers supplemented with 0.5 mL.L^−1^ drinking water. The results were consonant with those of Cai *et al*. [59] who used NS at a rate of 0.0, 0.3, 0.5, 1.0, or 2.0 mg/kg in 1-day-old Arbor broilers, they stated significant increase in the glutathione activity, malondialdehyde formation, and IgM concentrations. They also recommended 0.3:0.5 mg NS/kg as optimum supplementation levels with 1.0 mg NS/kg as maximum supplementation levels in broilers. Bakhshalinejad *et al*. [60] also supplemented broilers with 0.4 mg/kg NS for 1-day-old male Ross broilers and revealed significant enhancements in IgG concentrations. Xiao *et al*. [61] and Gulyas *et al*. [62] also recorded significant increases of immunoglobulin concentration in broilers supplemented with NS and they attributed the increase in the immunoglobulin levels to the enhancing influence of NS on protein synthesis and through increasing the eukaryotic translation initiation factor 5A-1, which contributed in the increase of protein synthesis, and thus increased IgG and M concentrations. Da Silva *et al*. [63] in synchronization with the current results reported that organic forms of Se significantly decreased antibody titer through the action on tissue and organ integrity and the enhancement in the metabolic rates. Boostani *et al*. [64] conducted a randomized trial to compare the influence of organic Se (Sel-Plex^®^), inorganic Se (sodium selenite), and NS (10:45 nm, 99.95% purity) in a rate of 0.3 mg/kg diet on 320 male Cobb^®^500 broilers in the presence and absence of oxidative stress, they recorded significant increases of IgG and IgM serum concentrations in broilers supplemented with NS compared to birds supplemented with Se in the presence of oxidative stresses.

Hubbard broilers in our study supplemented with 0.5 mL.L^−1^ drinking water NS revealed significant reductions in TBC and TEC rather than 1.0 mL NS and commercial Se supplementation. The results were supported by those of Barko *et al*. [65] who revealed that NS stimulate and encourage intestinal normal inhabitant to compete directly against opportunistic and pathogenic microorganisms for their receptor sites and prevent their growth and multiplication. Moreover, NS raised the level of specific immunity that contributed in a significant decline in total bacterial and TEC s. Gangadoo *et al*. [66], in agreement with our results, found that using NS in poultry might have direct antibacterial actions against pathogens as *E. coli*. Stanley *et al*. [67] recorded significant antimicrobial activities of NS against *E. coli*. Yip *et al*. [68] also in agreement confirmed the antimicrobial activity of NS against bacterial microorganisms such as *E. coli* and fungal organisms such as *Trichophyton rubrum*. Kheradmand *et al*. [69] recorded the antimicrobial action of NS against bacterial microorganisms such as *Pseudomonas aeruginosa* and fungal organisms such as *Candida albicans*. Shakibaie *et al*. [70] also recorded the antimicrobial actions of NS against *Proteus mirabilis*.

NS has gained much attraction for many reasons like its high bioavailability and low toxicity. The current study focused on more interest in the withdrawal time of the Se and NS from broilers’ organs and tissues. Thus the study revealed longer persistence of inorganic Se (sodium selenite) in broiler muscles and liver compared to NS. Electron microscopy images showed the disappearance of NS from the liver of broiler supplemented with 0.5 mL NS (0.05 mg.L^−1^) and its presence in the liver of broiler supplemented with 1.0 mL NS (0.1 mg.L^−1^) by the 20^th^ day. RP-HPLC showed that sodium selenite (0.5 and 1.0 mL) was detectable in both liver and muscles up to the 45^th^-day post-sacrificing, 0.5 mL NS disappeared in muscles at the 20^th^ day and in liver at the 30^th^-day, and 1.0 mL NS disappeared in muscles at 30^th^ day and in liver at 35^th^-day post-sacrificing. Mohapatra *et al*. [71] studied the influence of NS and inorganic Se (sodium selenite) in a rate of 0.3 mg/kg diet on Se bioavailability and deposition in the liver, breast muscles, pancreas, kidney, feathers, spleen, bursa of Fabricius, and thymus of 300 layer chicks up to 8^th^ week; they reported that Se contents in the organs and tissues under-study were significantly increased in broilers supplemented with NS rather than in inorganic Se. Wang [72] reported that NS could serve as another Se form and successfully improved tissue Se content of avian broilers compared with the control group. The current results confirmed that NS tissue residues disappeared earlier in the liver and muscles compared to Se residues that persist up to 45^th^-day samples. Similar results were recorded by Pan *et al*. [73] who found that liver, kidney, spleen, breast muscles, and whole-body Se concentrations in Rohman laying hens were higher in the groups given Se. Diskin *et al*. [74] and Hu *et al*. [75] reported a higher Se content in the liver than in muscle across all treatments. According to FSANZ [76], the limit of reporting (LOR) for Se was 0.01 mg/kg, which is convenient with NS in the current results rather than Se where the detected residues in both doses of Se were above the LOR. NHMRC [77] also reported that the recommended dietary intake (RDI) of Se for all subpopulations should not exceed 0.00179 mg/kg; so, the results of NS in the current study were below RDI within the two doses. The early disappearance of NS from liver and muscles compared to inorganic Se might be contributed to the nano-particles size (25 nm) and the characterizations that ensured minimum or no pathological affection in broilers supplemented with lower doses (0.5 mL 100 mg.L^−1^ equivalents to 0.05 mg.L^−1^ drinking water). Gandadoo *et al*. [78], Gangadoo *et al*. [66], and Shi *et al*. [79] confirmed that NS particles showed enhanced absorption, bioavailability, and rapid excretion from the body contributing less negative effect on tissues and organs.

Histopathological photomicrographs of the liver, spleen, bursa of Fabricius, and intestine, as well as immunohistochemistry photomicrographs of the duodenum in the current study, revealed enhanced and nearly normal tissue architecture in broilers supplemented with NS (0.5 and 1.0 mL) compared to broilers supplemented with Se and to un-supplemented control broilers even in the presence of *E. coli* infection. The recorded enhancement was consistent with those recorded by Alkhudhayri *et al*. [80] who reported improved histological tissue architecture from NS supplementation in the presence of some overwhelming challenges as *E. coli* infection. Mousa and Ali [81] also reported the enhanced influence of nanoparticles on the liver cellular architecture in face of *E. coli* infection in Africa ostrich chicks as well as the increased levels of resistance in the birds. The current results were also consistent with those recorded by Selim *et al*. [82] who recorded some adverse influences in liver histopathology such as inflammation and necrosis from supplementing broilers with higher doses of NS or Se compared to the influence of lower levels. The results also stated that Se-Yeast or Zn-Se-Meth produced higher levels of liver tissue safety. Attia *et al*. [83] also evaluated the influence of sodium selenite (inorganic Se) and Sel-plex (organic Se) at levels of 0.15 and 0.30 ppm compared to NS on the productive performance of Egyptian chicken strain (Gimmizah). They recorded mild influences produced by either NS or organic Se on hepatic tissues compared to inorganic Se.

## Conclusion

NS supplementation in Hubbard broilers revealed significant improvements in the productive performance, carcass quality, meat quality, and immunoglobulin concentration, as well as a significant antibacterial action regarding the usage of 0.5 mL NS at a rate of 100 mg/L NS (0.05 mg.L^−1^). The recorded enhanced measurements were aligned with determining zero residues in muscles and edible organs as well as, greater improvement in tissues and organs architecture.

## Authors’ Contributions

ESS designed the experimental design, supervised, and participated in the preparation and execution of the experiment, and in writing the manuscript. FFM conducted carcass quality parameters, pH and drip losses, and participated in writing the manuscript. MAF conducted HPLC and EM examination in liver and muscle samples and participated in writing the manuscript. RTH conducted the histopathological and immunohistochemistry examination and participated in writing the manuscript.
